# Th17 cells and IL-17A in traumatic brain injury: mechanisms and therapeutic perspectives

**DOI:** 10.3389/fimmu.2026.1850719

**Published:** 2026-08-28

**Authors:** Xin Guo, Jia Wang, Siyu Zhu, Boyu Sun, Longlong Tian, Mingkang Li, Shiyao Feng, Guozhu Sun

**Affiliations:** 1 Department of Neurosurgery, The Second Hospital of Hebei Medical University, Shijiazhuang, China; 2 Department of Neurosurgery, Xingtai Central Hospital, Xingtai, China; 3 Operations Management Office, The Second Hospital of Hebei Medical University, Shijiazhuang, China

**Keywords:** blood-brain barrier, IL-17A, IL-17A signaling, neuroimmune interaction, neuroinflammation, Th17 cells, traumatic brain injury

## Abstract

Traumatic brain injury (TBI) triggers a complex cascade of immune responses that extend beyond the central nervous system and contribute to secondary neuropathology. Increasing evidence implicates T helper 17 cells (Th17) and their signature cytokine interleukin-17A (IL-17A) in post-traumatic neuroinflammatory processes, including blood-brain barrier (BBB) disruption and neuroimmune interactions. This review synthesizes current evidence on the context-dependent roles of Th17 cells and IL-17A in TBI, with a focus on underlying molecular mechanisms, cellular targets, and the limitations of existing experimental data. We further discuss emerging therapeutic strategies aimed at modulating the Th17/IL-17A axis, as well as recent observations linking IL-17A signaling to neural plasticity and functional outcomes. Taken together, these findings highlight both the translational challenges and conceptual opportunities associated with targeting Th17/IL-17A-related pathways following TBI.

## 1 Introduction

Traumatic brain injury (TBI) is a heterogeneous neurological disorder caused by external mechanical forces and represents a major cause of mortality and long-term neurological disability worldwide, contributing substantially to years lived with disability (YLDs) (1, 2). The pathological progression of TBI is generally characterized by an immediate primary injury, followed by a complex secondary injury cascade that evolves over hours to months after the initial insult (3, 4). While primary injury involves direct mechanical damage to neural tissue, secondary injury encompasses multiple interconnected processes, including excitotoxicity, oxidative stress, mitochondrial dysfunction, blood-brain barrier (BBB) disruption, neuroinflammation, and chronic neurodegenerative changes (2, 4–7). These secondary injury mechanisms may persist for months or even years after injury and are increasingly recognized as important contributors to long-term neurological dysfunction, thereby representing potential targets for therapeutic intervention (6, 7). Among the secondary injury processes that follow TBI, neuroinflammation is widely regarded as a central driver of post-traumatic pathology and is characterized by the rapid activation of both immune and glial responses (2). BBB disruption further facilitates the infiltration of peripheral immune cells into the injured brain, amplifying inflammatory cascades (5). In parallel, leukocyte recruitment and metabolic disturbances have been observed throughout the evolution of injury, particularly over extended time courses (4, 6). Increasing evidence supports the view that TBI can act as a trigger for progressive neurodegenerative processes, linking acute injury to long-term structural and functional decline (8–10). Consistent with this concept, multi-omics studies have identified persistent immune dysregulation as a key molecular signature across both acute and chronic phases of TBI, underscoring the central role of neuroinflammation in shaping long-term outcomes (7).

While early neuroinflammatory responses after TBI have been predominantly attributed to innate immune activation, adaptive immune components are increasingly recognized as important contributors to the post-injury inflammatory milieu (7, 11). Among these, T-cell-mediated responses have attracted growing attention (12, 13). In particular, T helper 17 (Th17) cells and their signature cytokine interleukin-17A (IL-17A) are well-established immunomodulators with potent pro-inflammatory effects (14, 15). IL-17A has been shown to act on endothelial cells and influence BBB integrity within the central nervous system (CNS) (16), and has been implicated in excitotoxic and inflammatory mechanisms affecting neural cells in other disease contexts (17). In addition, IL-17A signaling can influence glial populations, including microglia and astrocytes, suggesting a broad role in CNS inflammatory regulation (18, 19).

Experimental studies have demonstrated the involvement of IL-17 signaling in secondary brain injury following TBI (20), and increased IL-17 expression has been observed in injured brain tissue in preclinical models (20). More broadly, neuroinflammation represents an important component of secondary injury and contributes to the evolving pathological cascade following TBI (21). Clinical investigations further support the presence of sustained neuroinflammatory activity after TBI, as reflected by elevated cerebrospinal fluid biomarkers and associations between immune-response profiles and neurological outcomes (22–24). Beyond TBI, IL-17A has been implicated in multiple CNS disease models, including processes related to BBB dysfunction, aberrant neurogenesis, and anxiety-like behavior (25–28). In experimental CNS injury models, IL-17A has also been reported to regulate ependymal cell proliferation and influence functional recovery in a context-dependent manner (29), while studies in neurodegenerative diseases such as Alzheimer’s disease further suggest potential effects on neural pathology (30). Collectively, these findings indicate that IL-17A may exert both pathogenic and potentially reparative effects depending on cellular context, signaling intensity, and temporal stage.

Th17/IL-17A-mediated immune pathways have been implicated across multiple inflammatory disease contexts (31–33), raising the possibility that similar mechanisms may also operate in TBI. Building on these observations, accumulating evidence suggests that Th17/IL-17A-related responses are involved in both central and peripheral immune alterations following TBI. These responses exhibit dynamic temporal changes and are associated with diverse cellular sources and inflammatory processes during the evolution of injury. Rather than representing a single linear pathogenic pathway, IL-17A signaling appears to participate in multiple aspects of the post-traumatic inflammatory milieu, although its precise role within broader neuroimmune responses remains incompletely defined. These considerations highlight the potential relevance of IL-17A-related pathways across different stages of post-injury progression, while underscoring the need for further studies to clarify their mechanistic contributions. This review synthesizes evidence identified through literature searches of PubMed and related databases, focusing on Th17/IL-17A signaling in TBI and related CNS disorders. It provides an integrated overview of the temporal and spatial dynamics and underlying molecular mechanisms of Th17/IL-17A responses across central and peripheral compartments, along with emerging therapeutic strategies and future research directions.

## 2 Fundamental biology of Th17 cells and IL-17A

### 2.1 Differentiation and stabilization of Th17 Cells

Th17 cells represent a distinct lineage of CD4^+^ T lymphocytes that arise in response to a cytokine environment enriched in IL-6, IL-1β, and TGF-β, with IL-23 contributing to stabilization and pathogenic maturation (34). This cytokine milieu induces key regulatory pathways involving the master transcription factor Retinoic acid receptor-related orphan receptor gamma t (RORγt) and Signal transducer and activator of transcription 3 (STAT3), which together define Th17 identity and effector function (35, 36). Emerging Th17 cells are typically characterized by the production of IL-17A, IL-17F, and in many settings, Granulocyte-macrophage colony-stimulating factor (GM-CSF), as well as surface expression of chemokine receptors such as CCR6 and, in some subsets, CXCR3 or CCR4, which together shape their migratory behavior and tissue tropism (37).

Following differentiation, IL-23 plays a key role in the maintenance and pathogenic function of Th17 cells (14). IL-23 signaling engages transcriptional pathways involving STAT3 and RORγt that are associated with Th17 pathogenicity (14, 38). In parallel, IL-23 promotes Th17 cell expansion and a pathogenic phenotype marked by IL-17A and GM-CSF production (35). Metabolic programming shapes Th17 fate and function: glycolysis favors pathogenic Th17 differentiation, whereas a shift toward oxidative phosphorylation restrains IL-17A production and promotes a more regulatory profile (39–42).

**Figure 1:**
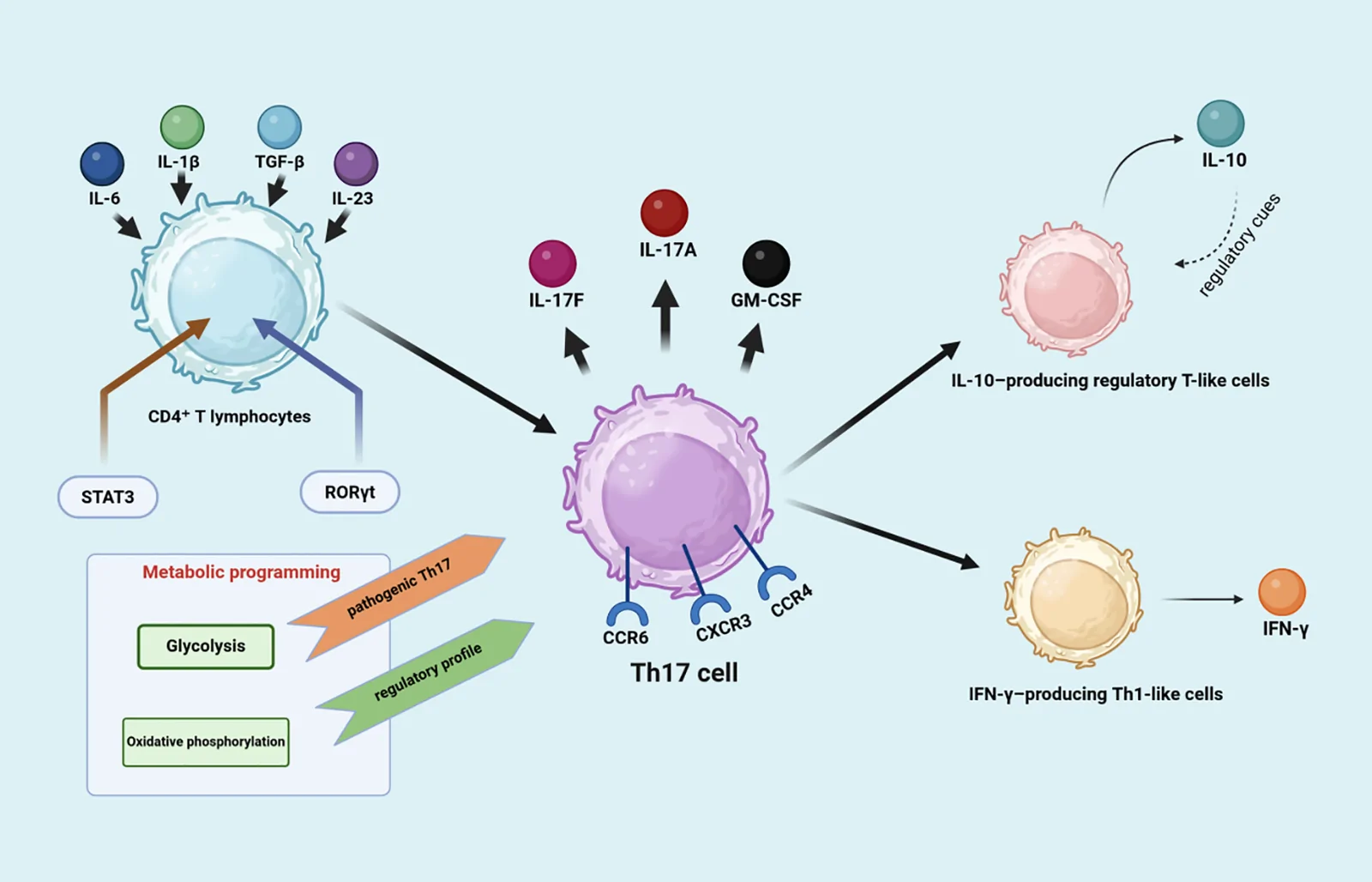
Differentiation, stabilization, and phenotypic plasticity of Th17 cells. Created in BioRender. Xin, G. (2026) https://BioRender.com/f1u07vi.

Increased IL-10 and exposure to regulatory cues have been associated with a functional shift of Th17 cells toward IL-10-producing or regulatory Th17-like states (43). Consistent with this notion, lineage-tracing and fate-mapping studies demonstrate that Th17 cells exhibit pronounced plasticity rather than representing a fixed differentiation endpoint, and can convert into IFN-γ-producing Th1-like cells or IL-10-producing regulatory T-like cells under changing cytokine conditions (33, 43, 44). This plasticity suggests that, in certain disease contexts, Th17 cells can adopt a spectrum of functional states ranging from pathogenic to regulatory, rather than representing a terminally fixed lineage (32). A schematic overview of the cytokine signals, transcription factors, metabolic features, and phenotypic plasticity involved in Th17 differentiation and stabilization is summarized in **Figure 1**.

Naïve CD4^+^ T lymphocytes differentiate into Th17 cells in response to cytokines including IL-6, IL-1β, and TGF-β, with IL-23 contributing to their stabilization and pathogenic maturation. These cytokine signals activate the lineage-defining transcription factors RORγt and STAT3. Th17 cells are characterized by the production of IL-17A, IL-17F, and GM-CSF, as well as the expression of chemokine receptors such as CCR6 and, in specific subsets, CXCR3 or CCR4. IL-23 contributes to the maintenance and pathogenic stabilization of Th17 cells. Metabolic programming further shapes Th17 fate, with glycolysis favoring pathogenic features and oxidative phosphorylation favoring less pathogenic or regulatory-like phenotypes. Th17 cells exhibit phenotypic plasticity and can acquire IFN-γ-producing Th1-like or IL-10-producing regulatory T-like characteristics under changing cytokine conditions.

### 2.2 General characteristics of IL-17A

IL-17A is a pro-inflammatory cytokine and the signature effector molecule of the Th17 lineage. Structurally, IL-17A belongs to the IL-17 cytokine family (IL-17A-F) and adopts a conserved cystine-knot fold, enabling the formation of IL-17A/A homodimers or IL-17A/F heterodimers (45, 46).

Although Th17 cells are a major source of IL-17A, this cytokine can also be produced by several innate-like lymphocyte populations, including γδ T cells, group 3 innate lymphoid cells (ILC3s), and natural killer T cells (33). Under inflammatory conditions, CNS-resident glial cells, particularly microglia, respond to IL-17A signaling and participate in inflammatory amplification (33, 47). This diversity of cellular sources allows IL-17A to function as a rapid-response cytokine that bridges innate and adaptive immunity. IL-17A signaling is mediated through a heteromeric receptor complex composed of IL-17RA and IL-17RC (48). Within the CNS, IL-17A-responsive cell populations include components of the neurovascular unit, such as endothelial cells, astrocytes, microglia, and neurons, suggesting that IL-17A signaling may influence multiple aspects of CNS inflammatory regulation (47, 49–52).

In these responsive cells, ligand engagement of the IL-17RA/IL-17RC receptor complex recruits the adaptor protein Act1 through SEFIR-SEFIR interactions, forming a signaling platform that engages TRAF6 and activates downstream Nuclear factor kappa B (NF-κB), Mitogen-activated protein kinases (MAPKs) (ERK1/2, p38, JNK), C/EBP transcription factors, and STAT3 (53, 54). These pathways induce the expression of inflammatory mediators, including chemokines (CXCL1, CXCL2, CXCL8), cytokines (IL-6, GM-CSF), and matrix-remodeling enzymes such as MMP-3 and MMP-9 (31, 53). A notable feature of IL-17A signaling is its strong synergy with other inflammatory mediators, particularly TNF-α, IL-1β, and IFN-γ, resulting in amplified transcriptional responses that have been described as the “IL-17 amplifier” effect (53, 55). These synergistic interactions, together with broad receptor expression and diverse cellular sources, establish IL-17A as an important driver of inflammatory responses and a potential therapeutic target in CNS disorders.

**Figure 2:**
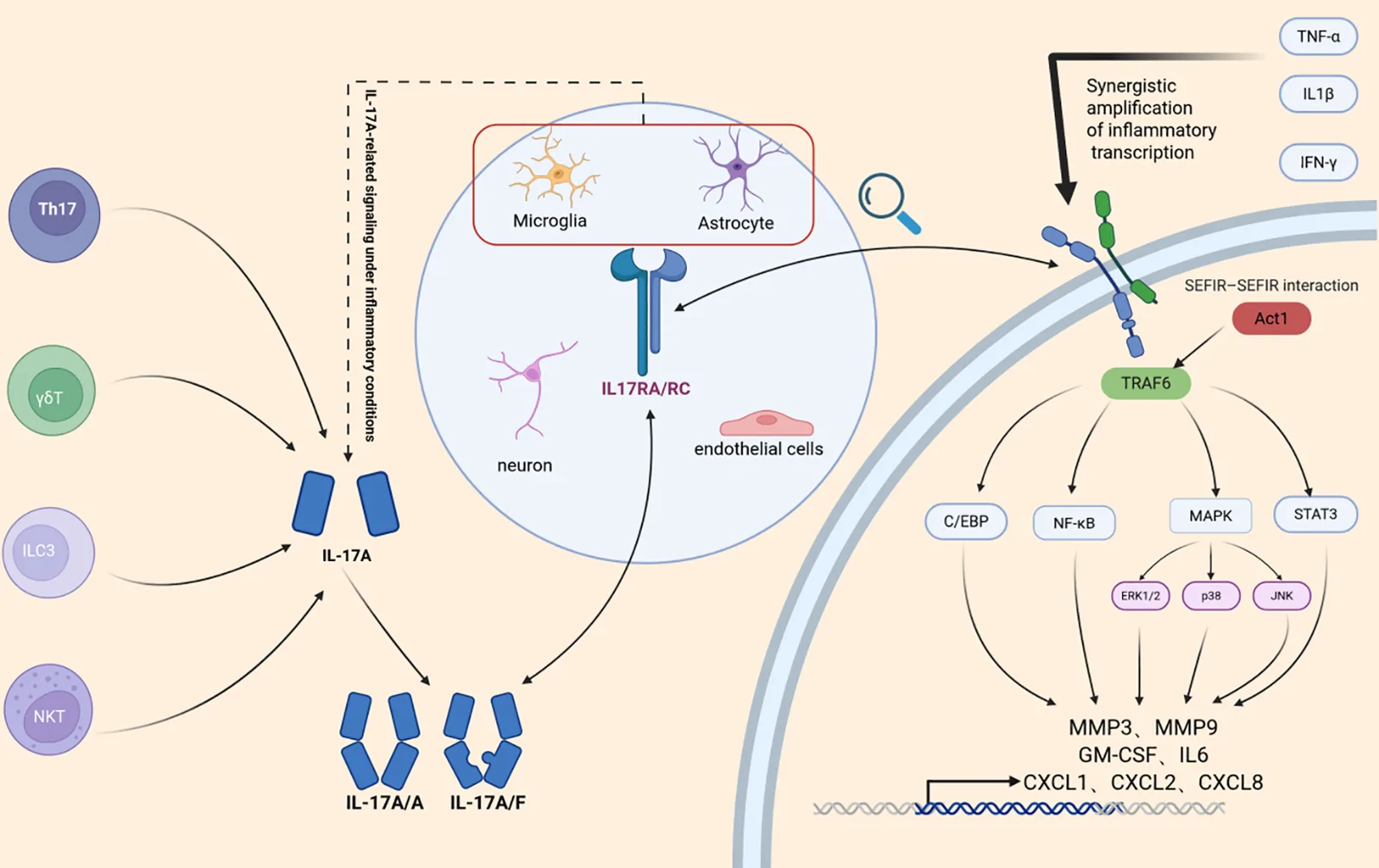
General characteristics and signaling mechanisms of IL-17A in the CNS. Created in BioRender. Xin, G. (2026) https://BioRender.com/7c6nwn2.

Collectively, these structural and signaling properties underpin the diverse functions of IL-17A in inflamed tissues and the CNS. **Figure 2** illustrates the major cellular sources, receptor interactions, cytokine synergy, and downstream signaling pathways associated with IL-17A.

IL-17A is a pro-inflammatory cytokine produced by Th17 cells and several innate-like lymphocyte populations, functioning as a rapid-response mediator bridging innate and adaptive immunity. IL-17A forms homodimeric (IL-17A/A) or heterodimeric (IL-17A/F) cytokine complexes that signal through the heterodimeric IL-17RA/IL-17RC receptor complex expressed by diverse neurovascular-unit cell types, including astrocytes, microglia, neurons, and endothelial cells. Receptor engagement recruits the adaptor Act1 via SEFIR-SEFIR interactions, leading to TRAF6-dependent activation of NF-κB, MAPKs, and C/EBP transcription factors, with additional downstream inflammatory signaling pathways such as STAT3 reported in certain contexts. In addition to direct receptor signaling, IL-17A strongly synergizes with other inflammatory cytokines, amplifying inflammatory gene expression programs and neuroinflammatory responses.

### 2.3 IL-17A production and distribution in the CNS

IL-17A signaling contributes to neuroinflammatory responses in the CNS by acting on multiple resident cell populations (56, 57). CNS-associated IL-17A may originate from infiltrating Th17 cells, meningeal γδ T cells, and other innate-like lymphoid populations (56–59). Following TBI, IL-17 levels increase rapidly in the injured CNS and, in some studies, in peripheral circulation, becoming detectable within hours and persisting for several days in experimental models (20). Consistent with this sustained neuroinflammatory activation, clinical CSF studies in concussion cohorts have reported prolonged elevation of inflammatory cytokine signatures in patients with persistent symptoms (22).

Single-cell RNA-sequencing studies of TBI highlight prominent inflammatory transcriptional remodeling in microglia and astrocytes (and, in some datasets, vascular/endothelial compartments), consistent with broad activation of cytokine-responsive programs that could include IL-17A-associated signaling in specific contexts (60, 61). IL-17A exerts pleiotropic pathological effects on vascular stability, glial activation, leukocyte recruitment, and neuronal signaling across the CNS, reflecting the broad expression of IL-17 receptors across multiple CNS-resident cell populations (47, 57, 62).

### 2.4 IL-17A function and its regulatory mechanisms

Beyond the canonical signaling pathways described above, IL-17A exerts broad immunomodulatory functions across diverse inflammatory settings. IL-17A plays an important role in coordinating immune responses at barrier surfaces and inflamed tissues (53, 63). It contributes to neutrophil-associated inflammation through the induction of neutrophil-attracting chemokines and regulation of granulopoietic pathways (63, 64). In addition, IL-17A acts on non-hematopoietic cells, including epithelial and stromal cells, to induce the expression of cytokines, chemokines, adhesion molecules, and matrix-remodeling enzymes, thereby amplifying local inflammatory responses (63–66).

IL-17A-related immune responses are regulated through coordinated transcriptional, post-transcriptional and receptor signaling mechanisms that fine-tune downstream inflammatory output (63–70). RNA-binding proteins such as Arid5a regulate inflammatory mRNA stability and can enhance IL-17-driven gene expression, whereas Act1-dependent ubiquitination facilitates TRAF6 recruitment and downstream signaling (68, 71, 72). Conversely, negative regulators including A20, TRAF3, and soluble IL-17RA isoforms serve to restrain IL-17 receptor signaling and prevent excessive inflammatory activation (73–75).

Extrinsic factors, including environmental cues, microbial signals, and metabolic programs, further influence the differentiation and function of IL-17A-associated immune responses (63, 76). Commensal microbiota and their metabolites contribute to immune homeostasis by regulating IL-17A-producing cell populations and maintaining the Th17/Treg balance (77–79). Moreover, local cytokine milieus and metabolic conditions can shape Th17 cell plasticity and functional heterogeneity in inflamed tissues (37, 43, 44). Collectively, these regulatory mechanisms determine the magnitude, duration, and tissue-specific effects of IL-17A-associated immune responses under inflammatory conditions.

## 3 Dynamic changes of Th17/IL-17A responses in TBI

TBI induces widespread neuroimmune dysregulation in the CNS and in peripheral compartments, and accumulating evidence implicates the Th17/IL-17A axis as an important contributor to secondary neuroinflammation (80). These changes unfold rapidly after injury and evolve dynamically over time, involving coordinated responses among resident glial cells, infiltrating lymphocytes, endothelial cells, and peripheral immune organs (7, 81, 82). Understanding these spatially and temporally distinct alterations is crucial for defining how IL-17A shapes acute and chronic neurological outcomes following TBI.

### 3.1 CNS changes of Th17/IL-17A after TBI

TBI triggers rapid neuroimmune activation within the injured cortex and hippocampus. Within hours after injury, resident microglia, astrocytes, and infiltrating myeloid cells upregulate pro-inflammatory cytokines such as IL-1β and IL-6; early increases in IL-23 have also been reported, together creating a cytokine milieu permissive for Th17 differentiation and IL-17A production (81, 83, 84). In parallel with this early inflammatory response, molecular signatures associated with Th17 polarization, including increased IL-17A expression and upregulation of Th17-related cytokines, are altered after injury (7, 85). In a rat TBI model, IL-17 expression in injured brain tissue increased as early as 6 h after injury and peaked at 3 days, accompanied by elevated IL-17 levels in both cerebrospinal fluid and serum, consistent with the evolving inflammatory response following TBI (20). BBB disruption occurs within hours after TBI and is associated with molecular changes that promote the subsequent recruitment of circulating immune cells into the injured CNS (7).

As the inflammatory response evolves over the ensuing days, the CNS undergoes dynamic changes in immune-cell trafficking. Flow-cytometric analyses in a mouse CCI model demonstrated that γδ T cells accumulated in the injured brain within the first few days after injury, increased markedly by day 3, and peaked around day 7, representing an early source of IL-17A during acute neuroinflammation (86). Conventional CD4^+^ Th17 cells, another major source of IL-17A, may also participate in IL-17A-associated immune responses within the injured CNS (87). In a mouse CCI model, Th17 polarization was significantly increased in both the injured brain and peripheral blood as early as 24 h after injury, remained elevated at 72 h, and persisted for up to 3 weeks following trauma (88).

During later stages of injury, MHC-II-positive activated microglia were detected from 1 week after injury and persisted for up to 1 year following trauma, indicating prolonged activation of antigen-presenting pathways within the injured CNS (89). The same rat TBI study also demonstrated increased IL-23 expression within injured brain tissue, consistent with activation of the IL-23/IL-17 signaling axis following TBI (20). Given the established role of IL-23 in promoting the maintenance and pathogenic functions of Th17 cells (51, 52, 90), the persistence of MHC-II-positive microglia together with increased IL-23 expression suggests that molecular signals associated with antigen presentation and Th17-related immune responses may remain present across multiple stages of injury evolution.

In the weeks to months following injury, persistent neuroinflammatory activity has been reported in both experimental models and clinical studies, including chronic microglial activation and ongoing white-matter pathology that remain detectable long after the initial insult (7, 82, 89). Together with the sustained presence of Th17/IL-17A-associated immune signals observed during earlier stages of injury (20, 86, 88), these findings highlight a potential link between IL-17A-related immune responses and long-term post-traumatic neuroinflammation. However, the precise contribution of IL-17A to chronic neuroinflammatory remodeling after TBI remains to be fully elucidated.

### 3.2 Peripheral immune changes associated with Th17/IL-17A after TBI

Following TBI, systemic immune activation extends beyond the CNS. Within hours to days after injury, circulating pro-inflammatory mediators such as IL-6 and IL-1β increase (7). In a severe rat TBI model, immediate splenectomy reduced circulating IL-1β, TNF-α, and IL-6 levels at multiple time points between 6 h and 3 days after injury, suggesting that the spleen contributes to early systemic inflammatory responses following TBI (91). Because IL-6 and IL-1β are key cytokines involved in Th17 differentiation (92), this early systemic inflammatory milieu may create conditions favorable for Th17-associated immune responses.

Beyond splenic involvement, emerging evidence suggests that acute brain injury can also influence bone marrow hematopoiesis. Although derived from an intracerebral hemorrhage model, hematopoietic stem-cell activity and myeloid-lineage differentiation increased rapidly after injury, peaked around day 3, remained elevated through day 7, and declined toward baseline by day 14 (93). Because inflammatory mediators produced by myeloid cells are known regulators of Th17 differentiation (92), these hematopoietic changes may indirectly contribute to immune conditions permissive for Th17-associated responses.

Clinical studies have reported alterations in gut microbial composition within 72 h after severe trauma, characterized by reductions in beneficial bacterial populations and increases in Clostridiales and Enterococcus (94). In experimental TBI models, significant changes in gut microbial diversity and taxonomic composition were observed during the first week after injury and remained detectable at 35 days post-injury, accompanied by evidence of altered intestinal integrity (95). Given the established role of the gut microbiota in regulating immune-cell differentiation and inflammatory responses, including pathways involved in Th17 polarization (96), these post-traumatic alterations in microbial composition may have broader immunological consequences beyond the intestine.

Clinically, elevated circulating IL-17A levels have been reported in TBI patients during the days-to-weeks period following injury, with serial serum sampling over an approximately 2-week observation period demonstrating persistently higher IL-17A levels than those observed in non-TBI trauma controls (97). At later chronic stages, a longitudinal study of military personnel with blast-related TBI performed serum cytokine profiling at a mean of approximately 10 months after injury and identified persistent alterations in systemic cytokine networks (98). Notably, although IL-17 was included among the cytokines analyzed, no significant long-term changes were detected, indicating that current evidence for sustained chronic IL-17A dysregulation after TBI remains limited (98).

Peripheral IL-17A-associated immune responses may also influence CNS inflammation. Experimental studies using cultured brain endothelial cells have shown that IL-17A stimulation increases endothelial expression of adhesion molecules and chemokines, including VCAM-1, CCL2, and CXCL1, thereby promoting leukocyte adhesion and recruitment across the BBB (99). In addition, infiltrating neutrophils derived from the peripheral circulation have been identified as a major source of IL-17A within the injured brain during the subacute phase after TBI (100). These findings suggest that peripheral immune alterations may contribute to intracranial inflammatory processes through both leukocyte recruitment and local IL-17A production within the injured CNS.

**Figure 3:**
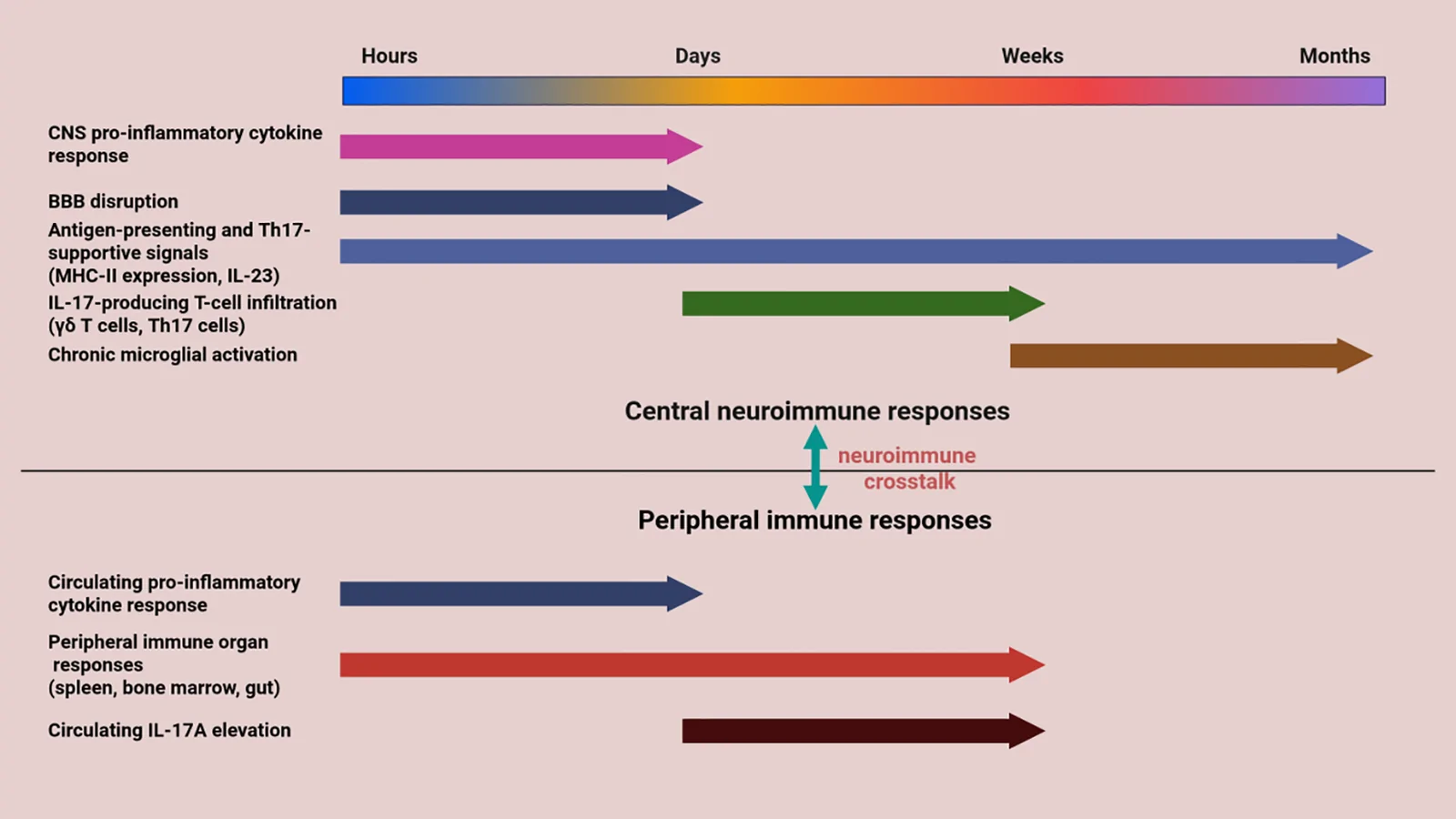
Temporal dynamics of CNS and peripheral Th17/IL-17A-associated immune responses following traumatic brain injury (TBI). Created in BioRender. Xin, G. (2026) https://BioRender.com/y4nudy2.

Collectively, these findings highlight the dynamic and interconnected nature of Th17/IL-17A-associated immune responses across both CNS and peripheral compartments following TBI (7, 81). The temporal dynamics of these responses are summarized in **Figure 3**.

TBI induces rapid neuroimmune activation in both the central nervous system (CNS) and peripheral immune compartments that evolves dynamically over time. Within hours after injury, a CNS pro-inflammatory cytokine response (including IL-1β, IL-6, IL-23, and IL-17A) is initiated in the injured brain, accompanied by blood-brain barrier (BBB) disruption. During the ensuing days to weeks, IL-17A-producing T lymphocytes, including γδ T cells and Th17 cells, infiltrate the injured tissue. In parallel, antigen-presenting and Th17-supportive signals, represented by increased MHC-II expression and IL-23-associated responses, emerge and may persist across multiple stages of injury evolution. During later phases, persistent neuroinflammatory activity is associated with chronic microglial activation and long-term neuropathological changes, processes that may be influenced by sustained IL-17A-related immune signaling.

In parallel, peripheral immune alterations occur, including a circulating pro-inflammatory cytokine response, activation of peripheral immune organs (spleen, bone marrow, and gut), and elevated circulating IL-17A levels. Horizontal arrows indicate the approximate onset and persistence of each process after TBI. Temporal patterns summarized here are derived from experimental TBI models and available clinical observations. The schematic highlights the dynamic interplay between CNS and peripheral immune responses across different stages of TBI progression.

## 4 Molecular mechanisms of Th17/IL-17A-mediated pathology in TBI

These immune alterations provide the biological context in which IL-17A-mediated mechanisms may contribute to secondary injury after TBI. Neuroinflammation is a defining pathological component of TBI and a major determinant of secondary tissue damage (7, 9). Acute disruption of the neurovascular unit following trauma alters immune communication between the CNS and the periphery, leading to sustained glial activation, leukocyte infiltration, and persistent neuroimmune dysregulation (6, 7). Within this inflammatory milieu, emerging evidence suggests that IL-17A-related signaling contributes to post-traumatic neuroinflammation, although much of the mechanistic framework is also informed by studies in other CNS diseases (100, 101). IL-17A can act on multiple neurovascular unit-related cell populations, including endothelial cells, glia, and neurons. In TBI, direct evidence is strongest for inflammatory amplification, whereas evidence for some cell-specific effects is primarily derived from studies in other CNS disorders (99, 101). The following subsections summarize the principal mechanisms through which Th17 cells and IL-17A may contribute to neuroinflammation, neurovascular dysfunction, and secondary injury processes after TBI.

### 4.1 IL-17A and blood-brain barrier dysfunction

TBI induces early BBB disruption, which facilitates peripheral immune cell extravasation and contributes to secondary injury cascades (6, 102). Mechanical trauma disrupts the neurovascular unit, causing endothelial injury and tight-junction breakdown. Concurrent activation of matrix metalloproteinases further degrades BBB components, collectively increasing vascular permeability after TBI (6, 103).

IL-17A may participate in the progression of BBB dysfunction after TBI through effects on both endothelial activation and barrier integrity. Experimental studies using cultured murine brain endothelial cells demonstrated that IL-17A stimulation increased VCAM-1 expression and induced the production of CCL2 and CXCL1, thereby promoting leukocyte adhesion and recruitment and creating a pro-inflammatory vascular environment that may facilitate immune-cell infiltration into the injured CNS (99). In addition, IL-17A has been reported to disrupt tight-junction organization, including alterations in claudin-5, occludin, and ZO-1 localization, thereby increasing BBB permeability in experimental BBB models (104). Among the immune-cell populations that may gain access to the injured CNS, Th17 cells are of particular interest because of their capacity to produce IL-17A. Th17 cells are characterized by the expression of CCR6 (92). In experimental TBI, CCL20 expression is increased in the injured brain during the acute post-traumatic period (105). Because CCL20 is the cognate ligand for CCR6, activation of the CCL20-CCR6 axis may provide a potential mechanism for recruiting Th17 cells into the injured CNS. However, direct experimental evidence demonstrating functional CCL20-CCR6-dependent Th17 trafficking across the BBB after TBI remains limited (105).

Collectively, these findings suggest that IL-17A may contribute to BBB dysfunction after TBI by promoting endothelial activation and disrupting barrier integrity. The resulting increase in immune-cell infiltration may further amplify secondary neuroinflammatory cascades within the injured CNS, potentially contributing to ongoing tissue damage and neurological dysfunction following trauma (100, 106).

### 4.2 IL-17A and activation of microglia and astrocytes

Microglia and astrocytes are the principal immune-responsive cells in the CNS and are rapidly activated after TBI. Activated microglia and reactive astrocytes produce pro-inflammatory cytokines and chemokines that promote leukocyte recruitment and contribute to the maintenance of post-traumatic neuroinflammation (7, 9).

A potential interaction between activated microglia and Th17/IL-17A-associated immune responses may contribute to the amplification of post-traumatic neuroinflammation. Microglia are among the earliest resident immune cells activated after injury and release numerous inflammatory mediators, including IL-1β, IL-6, TNF-α, chemokines, reactive oxygen species, and nitric oxide, which contribute to secondary neuronal injury and neurodegeneration (7, 9, 81). Notably, IL-1β and IL-6 are key cytokines involved in Th17 differentiation and IL-17A production (92), suggesting that microglia-derived inflammatory signals may help establish an immune environment permissive for Th17-associated responses after TBI.

Accumulating evidence from experimental CNS disease models further suggests that IL-17A can enhance microglial inflammatory activity. In OGD/reperfusion models, IL-17A knockdown attenuated microglial inflammatory responses and implicated ROS/HMGB1, p53, and PI3K/Akt signaling pathways (47). Similarly, IL-17A promoted microglia-dependent neuroinflammation and neuronal injury in experimental Parkinson’s disease models (49). In LPS-induced neuroinflammation, IL-17A neutralization reduced microglial activation and decreased TNF-α, IL-6, iNOS, and COX-2 expression, whereas recombinant IL-17A directly stimulated microglial inflammatory responses *in vitro* (56). Together, these observations suggest a potential microglia-Th17/IL-17A feedback loop in which microglial inflammatory mediators favor Th17/IL-17A-associated responses, while IL-17A in turn amplifies microglia-driven inflammation.

In addition to this potential microglia-Th17/IL-17A feedback loop, astrocytes may serve as important amplifiers of IL-17A-mediated neuroinflammatory responses within the CNS (50, 57). Reactive astrocytes are important sources of chemokines that regulate leukocyte recruitment within injured CNS tissue (7, 81, 107). Experimental evidence from Th17-mediated CNS inflammatory models suggests that IL-17A signaling in astrocytes contributes to inflammatory amplification. In EAE models, astrocyte-specific disruption of Act1 signaling reduced IL-17-dependent induction of chemokines and inflammatory mediators, including CXCL1, CXCL2, and CCL20, and was accompanied by decreased recruitment of lymphocytes, neutrophils, and macrophages into the CNS (50). Similarly, CNS-targeted IL-17A expression under the GFAP promoter induced astrocytosis, enhanced microglial activation, and exacerbated inflammatory responses, supporting a role for astrocytes as important effectors of IL-17A-mediated neuroinflammation (57). Collectively, these findings suggest that IL-17A can promote chemokine-mediated leukocyte recruitment and inflammatory amplification through astrocyte-dependent mechanisms.

Taken together, available evidence indicates that IL-17A may influence post-traumatic neuroinflammation through multiple glial-cell-mediated pathways. While activated microglia may participate in a microglia-Th17/IL-17A feed-forward interaction, astrocytes may amplify inflammatory signaling through chemokine production and leukocyte recruitment. These observations collectively suggest that IL-17A functions as a potential amplifier of glia-driven neuroinflammatory responses after TBI, although the precise cellular and molecular interactions involved remain incompletely characterized.

### 4.3 Potential roles of IL-17A in peripheral immune-brain communication

In addition to local CNS immune responses, accumulating evidence suggests that peripheral immune organs, including the spleen, gastrointestinal tract, and bone marrow, undergo substantial post-traumatic remodeling and may participate in bidirectional communication between the peripheral immune system and the injured brain (7).

As discussed above, the spleen contributes to early systemic inflammatory responses following TBI and serves as an important component of post-traumatic peripheral immune remodeling (91). Splenic inflammatory activity is associated with increased production of cytokines such as IL-1β, TNF-α, and IL-6 (91), which are recognized regulators of adaptive immune responses, including pathways involved in Th17-cell differentiation and IL-17A production (92). These observations suggest that splenic immune responses may contribute to the peripheral inflammatory milieu that supports Th17/IL-17A-associated immunity after TBI.

TBI also induces persistent alterations in gut microbial composition and intestinal homeostasis (94, 95). Experimental evidence further suggests that these changes may be mediated, at least in part, through neuroimmune and autonomic mechanisms linking brain injury to gut dysfunction (108). Given the established role of the gut microbiota in regulating systemic immunity and Th17-cell differentiation (96), post-traumatic dysbiosis may influence IL-17A-associated immune responses through the gut-brain axis. Supporting this concept, a recent study demonstrated that neutropenia-associated alterations in the gut microbiota promoted T-cell-derived IL-17A production, leading to increased G-CSF expression, enhanced granulopoiesis, and accelerated neutrophil recovery during hematopoietic stress. Antibiotic-mediated microbiota depletion suppressed IL-17A and G-CSF production, whereas fecal microbiota transplantation restored IL-17A-dependent granulopoietic responses, highlighting a functional link between gut microbiota, IL-17A signaling, and peripheral immune remodeling (109). Although direct evidence linking gut dysbiosis to Th17/IL-17A-associated immune responses after TBI is currently lacking, these findings suggest a potential link between microbiota alterations, IL-17A signaling, and peripheral immune remodeling following brain injury.

In parallel, TBI induces substantial alterations in bone marrow hematopoiesis and promotes the expansion of peripheral myeloid populations (93, 110, 111). Because expanded myeloid populations are important sources of inflammatory mediators involved in Th17 differentiation and maintenance (92), post-traumatic bone marrow remodeling may indirectly influence Th17/IL-17A-associated immune responses. Conversely, IL-17A has been implicated in the regulation of G-CSF-dependent granulopoiesis and neutrophil homeostasis in non-TBI settings (109, 112). Together, these observations raise the possibility of bidirectional interactions between bone marrow remodeling and IL-17A-associated immunity following TBI; however, a direct causal relationship has yet to be established.

Collectively, available evidence suggests that TBI-induced remodeling of peripheral immune organs may influence neuroinflammation through interconnected spleen-brain, gut-brain, and bone marrow-brain pathways. Within this framework, IL-17A may represent a potential mediator linking peripheral immune activation, microbiota-dependent immune regulation, hematopoietic adaptation, and neuroinflammatory responses after brain injury. However, the existence of a unified IL-17A-driven peripheral-central immune circuit integrating splenic, gut, and bone marrow responses after TBI has yet to be established.

### 4.4 IL-17A and neuronal dysfunction and network instability

Neuronal dysfunction and network instability are important components of secondary injury after TBI and contribute to cognitive impairment, behavioral abnormalities, and seizure susceptibility (3). In addition to indirect effects mediated through glial activation and neuroinflammation, accumulating evidence suggests that IL-17A may directly influence neuronal function.

To date, the strongest evidence for direct neuronal effects of IL-17A comes from studies examining synaptic plasticity. Experimental studies using primary hippocampal neurons and hippocampal slices demonstrated that neurons express IL-17RA and respond directly to IL-17A stimulation (113). IL-17A impaired long-term potentiation through IL-17RA-dependent activation of p38 MAPK signaling, indicating a direct effect on synaptic plasticity (113). Because synaptic plasticity is essential for learning, memory, and adaptive circuit remodeling after injury, these findings suggest that IL-17A may contribute to post-traumatic neuronal dysfunction by disrupting synaptic signaling pathways.

Beyond its effects on synaptic plasticity, IL-17A may also influence neuronal excitability. In a chemotherapy-induced neuropathic pain model, IL-17/IL-17R signaling enhanced excitatory postsynaptic currents, suppressed inhibitory postsynaptic currents and GABA-induced currents in spinal somatostatin-expressing neurons, and blockade or knockdown of IL-17R reduced neuronal hyperexcitability (114). In addition, evidence from autoimmune and neuroinflammatory CNS disorders suggests that Th17/IL-17A-associated inflammation is linked to cognitive impairment, synaptic dysfunction, neuronal injury, and disease progression in disorders such as multiple sclerosis, Parkinson’s disease, and Alzheimer’s disease, further supporting a role for IL-17A-related immune responses in the regulation of neuronal function under inflammatory conditions (115). These findings suggest that IL-17A may shift neural circuits toward increased excitability under inflammatory conditions; however, a direct contribution of IL-17A to excitotoxic neuronal injury after TBI has yet to be established.

Consistent with this concept, clinical studies in epilepsy have reported elevated serum and cerebrospinal fluid IL-17A levels, and higher IL-17A expression was associated with seizure severity and frequency (116). These observations support a potential association between IL-17A-associated inflammation and network hyperexcitability. More broadly, studies in epilepsy and neuroinflammatory disorders have shown that inflammatory mediators released during neuroinflammation can directly modulate neuronal excitability and synaptic transmission through effects on glutamatergic and GABAergic signaling pathways, thereby contributing to network instability and seizure generation (117). Although a direct role for IL-17A in post-traumatic epileptogenesis has yet to be established, these findings raise the possibility that IL-17A-associated inflammation may influence seizure susceptibility and neural circuit stability after brain injury. Furthermore, electrophysiological studies in experimental TBI models demonstrated impaired hippocampal long-term potentiation and altered network excitability following injury, changes that have been proposed to contribute to post-traumatic cognitive deficits and epileptogenesis (118).

Taken together, available evidence suggests that IL-17A may contribute to neuronal dysfunction through both direct and indirect mechanisms. Direct neuronal IL-17RA signaling can impair synaptic plasticity and increase neuronal excitability, whereas IL-17A-associated neuroinflammation may further compromise neural circuit stability through interactions with glial and immune pathways. Although most mechanistic evidence is derived from non-TBI models, these findings support a potential role for IL-17A in linking neuroinflammation to network instability, cognitive dysfunction, and seizure susceptibility following TBI.

### 4.5 IL-17A and chronic neurodegeneration after TBI

TBI is increasingly recognized as a chronic and progressive disease process rather than an isolated neurological event. Accumulating evidence indicates that neuroinflammatory responses initiated during the acute phase of injury may persist for months or even years and contribute to ongoing neurodegenerative changes following TBI (119). Chronic pathological alterations after TBI include sustained microglial activation, white-matter degeneration, progressive brain atrophy, disruption of structural and functional connectivity, and an increased risk of later neurodegenerative disorders (9, 119–121).

Persistent neuroinflammation is considered a major contributor to these long-term pathological processes. As discussed above, activated microglia can remain chronically reactive after TBI and continue to produce inflammatory mediators that promote tissue injury and neurodegeneration (9). Given the potential feed-forward interaction between microglia and Th17/IL-17A-associated immune responses described in Section 4.2, persistent IL-17A signaling may represent one mechanism by which acute inflammatory responses are sustained after TBI. Whether such sustained IL-17A-associated inflammation contributes directly to chronic neurodegenerative changes after TBI remains unclear.

Chronic inflammatory environments have also been implicated in impaired tissue repair and regenerative capacity within the injured CNS. Experimental studies have demonstrated that neuroinflammation can suppress adult hippocampal neurogenesis and alter the neural progenitor-cell niche, thereby limiting endogenous repair mechanisms (122). More specifically, IL-17 has been identified as a negative regulator of adult hippocampal neurogenesis; genetic deletion of IL-17 increased the number of newborn neurons in the dentate gyrus and enhanced the expression of proneuronal genes in neural progenitor cells (28). Persistent inflammation also promotes astrocyte reactivity and glial scar formation, processes associated with impaired axonal regeneration and long-term structural remodeling after CNS injury (123). Direct evidence linking IL-17A to glial scar formation after TBI has yet to be established. However, given its ability to promote reactive astrocyte responses and inflammatory amplification, IL-17A-associated signaling may contribute to chronic astrocytic remodeling, a possibility that warrants further investigation.

The cumulative impact of chronic neuroinflammation, impaired repair mechanisms, and progressive tissue degeneration is associated with long-term cognitive decline, affective disturbances, and an increased risk of neurodegenerative disorders following TBI (119, 124). Taken together, available evidence suggests that persistent IL-17A-associated inflammatory signaling may represent a potential link between acute post-traumatic immune activation and chronic neurodegenerative changes following brain injury. However, direct mechanistic studies defining the contribution of IL-17A to chronic neurodegeneration after TBI remain limited and warrant further investigation.

## 5 Therapeutic targeting of the Th17/IL-17A axis in TBI

In this section, we summarize current and emerging therapeutic strategies targeting the Th17/IL-17A axis in TBI, ranging from direct cytokine blockade to upstream immunomodulation and advanced delivery platforms.

Accumulating experimental evidence suggests that IL-17A-related (type 17) immune signaling contributes to post-traumatic neuroinflammation, motivating interest in therapeutic strategies targeting IL-17A activity or pathogenic Th17 responses (100). Therapeutic approaches can be broadly categorized into direct inhibition of IL-17A/IL-17RA signaling, modulation of pathways involved in Th17 differentiation and maintenance, and strategies designed to enhance delivery of immunomodulatory agents to the injured CNS (125, 126). In parallel, broader immune-modulating interventions aimed at restoring post-traumatic immune homeostasis are also being explored.

Collectively, these approaches represent an evolving therapeutic landscape (**Table 1**). However, most current strategies are supported primarily by preclinical evidence, and their clinical applicability in TBI has not yet been determined. Further translational and clinical studies are required to clarify whether targeting the Th17/IL-17A axis can improve long-term neurological and functional outcomes after TBI (127).

**Table 1:** Therapeutic targeting of the Th17/IL-17A axis in TBI.

Strategy	Model/context	Mechanistic rationale	Key observations	Evidence level	References
Direct IL-17A/IL-17RA blockade	Rodent TBI; γδ T cells; microglial inflammatory programs	Block IL-17A/IL-17RA signaling and downstream NF-κB, MAPK, and C/EBP pathways	Biological rationale established; functional benefit in TBI remains unproven	Preclinical TBI	(63, 86, 128, 129)
Approved IL-17A biologics (secukinumab, ixekizumab)	Clinical autoimmune diseases	Systemic neutralization of IL-17A	Clinically effective in autoimmune diseases; translational potential in TBI is supported by preclinical evidence but remains untested clinically	Clinical non-TBI	(130)
IL-23 p19-directed blockade	Autoimmune and neuroinflammatory models	Inhibit IL-23-dependent stabilization and pathogenic maturation of Th17 cells	Suppresses Th17 expansion and IL-17A production in non-TBI models	Preclinical non-TBI	(90, 131)
RORγt inhibition	Preclinical autoimmune models	Suppress Th17 differentiation through inhibition of the lineage-defining transcription factor	Reduces pathogenic Th17 responses; efficacy in TBI unknown	Preclinical non-TBI	(67, 132)
Upstream cytokine modulation (IL-6, IL-1β)	Th17 differentiation and maturation pathways	Indirectly reduce IL-17A production by targeting upstream cytokine networks	May attenuate downstream IL-17A signaling by limiting Th17 differentiation and expansion; evidence in TBI remains limited.	Conceptual	(14, 90)
Promotion of Th17 plasticity	Th17-cell lineage plasticity	Promote transition toward regulatory or less pathogenic phenotypes	Potential immunoregulatory benefit through resolution of chronic inflammation; not evaluated in TBI	Conceptual	(133)
Nanoparticle-based delivery platforms	CNS drug delivery	Enhance BBB penetration, targeted CNS delivery, and sustained release of therapeutic agents	Enabling technology that may improve delivery of IL-17A-targeted therapies; IL-17A-specific efficacy in TBI remains untested	Platform-based	(134–139)
Restoration of immune homeostasis (Tregs, gut microbiota, systemic immune regulation)	Peripheral immunity and gut-brain axis	Enhance regulatory immunity and normalize immune balance	Supported by associative and experimental evidence; direct causal effects in TBI remain uncertain	Preclinical/indirect	(77, 110, 140–142)

This table summarizes current and emerging therapeutic strategies targeting the Th17/IL-17A axis in TBI, as discussed in Section 5. Strategies are grouped according to their primary biological target or functional role. The evidence categories indicate the source and context of supporting evidence (e.g., clinical, preclinical, conceptual, indirect, or platform-based) rather than a formal hierarchy of therapeutic efficacy. Several approaches remain exploratory and are currently supported primarily by non-TBI or mechanistic studies.

### 5.1 Direct inhibition of IL-17A and IL-17RA

Targeting IL-17A/IL-17RA has been proposed as a potential immunomodulatory strategy in TBI; however, direct preclinical evidence supporting neuroprotection through IL-17A pathway blockade remains limited (128). In experimental TBI, IL-17-associated immune responses, including IL-17 production by infiltrating γδ T cells, can influence microglial inflammatory programs, providing a biological rationale for targeting this pathway. Nevertheless, direct evidence demonstrating that IL-17A blockade reduces lesion burden or improves neurological recovery remains sparse (86, 128, 129). Mechanistically, IL-17RA signaling activates canonical downstream pathways including NF-κB, MAPKs, and C/EBP transcriptional programs, suggesting that pathway inhibition could attenuate inflammatory amplification (63).

Although IL-17A inhibitors (e.g., secukinumab and ixekizumab) have been approved for autoimmune diseases such as psoriasis and ankylosing spondylitis, their application in TBI remains unexplored clinically (130). Given broader evidence implicating neuroinflammatory cytokine signaling in seizure susceptibility and epileptogenesis, modulation of IL-17A-related pathways may potentially influence post-traumatic epileptogenesis. However, direct evidence supporting IL-17A inhibition as a strategy to prevent post-traumatic epilepsy is currently lacking (116).

Future studies should also consider the context-dependent functions of IL-17A in the CNS, as complete pathway inhibition may not uniformly confer benefit across different stages of injury (128). Together, these observations place direct IL-17A/IL-17RA blockade as a biologically rational but still experimentally evolving therapeutic strategy in TBI.

### 5.2 Modulation of Th17 differentiation and pathogenic maturation

Beyond direct cytokine inhibition, several therapeutic strategies aim to modulate upstream pathways that govern Th17 differentiation, stabilization, and pathogenic maturation.

Given the essential role of IL-23 in stabilizing and licensing pathogenic Th17 cells, therapeutic targeting of IL-23, particularly through p19-directed blockade, has emerged as an attractive upstream approach for modulating the Th17/IL-17A axis (90). Experimental studies in autoimmune and neuroinflammatory disease models have demonstrated that disruption of IL-23 signaling suppresses Th17 expansion, reduces IL-17A production, and attenuates CNS inflammatory pathology (131). However, direct evidence supporting these effects in TBI models is currently lacking.

Pharmacological inhibition of RORγt, the lineage-defining transcription factor of Th17 cells, represents another selective strategy for suppressing pathogenic Th17 responses (67, 132). Small-molecule RORγt inhibitors have demonstrated anti-inflammatory activity across multiple autoimmune disease models by reducing Th17 differentiation and limiting Th17-associated inflammatory programs (132). Whether similar approaches can effectively modulate post-traumatic neuroinflammation remains to be determined.

Additional cytokines involved in Th17 differentiation and pathogenic maturation, including IL-6 and IL-1β, have also been identified as important regulatory nodes within the Th17 developmental network (14). Modulation of these cytokine pathways may indirectly reduce IL-17A production and attenuate downstream inflammatory signaling within the CNS (90). Given the inherent plasticity of Th17 cells, future immunomodulatory strategies may also seek to promote transitions toward regulatory or less pathogenic phenotypes rather than simply suppressing Th17-cell activity (133). Such approaches could potentially contribute to the restoration of immune homeostasis following CNS injury.

Taken together, modulation of pathways that regulate Th17 differentiation, stabilization, and pathogenicity represents a promising upstream strategy for targeting type 17 immune responses. Although most supporting evidence is derived from autoimmune and neuroinflammatory disease models, these approaches provide a conceptual framework for future investigation of Th17-directed therapies in TBI.

### 5.3 Nanoparticle-based IL-17A-targeted therapies

One of the major challenges in developing cytokine-targeted therapies for TBI is efficient drug delivery across the BBB. The BBB excludes nearly all large biologics, including monoclonal antibodies, and severely restricts the penetration of most systemically administered therapeutics, thereby limiting CNS drug exposure despite adequate peripheral drug levels (134). Advances in nanomedicine have enabled the development of targeted and sustained drug-delivery platforms, raising the possibility that cytokine-directed therapies, including those targeting IL-17A-related pathways, could be adapted for nanoparticle-based CNS delivery (135).

Among currently available nanocarriers, poly(lactic-co-glycolic acid) (PLGA)-based nanoparticles have received particular attention because of their biocompatibility, biodegradability, and capacity for controlled drug release (136). Surface modification with polyethylene glycol (PEG), targeting peptides, or receptor-binding ligands can further enhance BBB transport and improve CNS accumulation of therapeutic cargos (135, 136). Although IL-17A-targeted formulations have not yet been evaluated in TBI, such delivery systems may provide a platform for improving brain exposure of anti-IL-17A biologics and other immunomodulatory agents.

Supporting the therapeutic potential of nanoparticle-based approaches, galantamine-loaded PLGA nanoparticles improved locomotor recovery in a rat spinal cord injury model and reduced oxidative stress, IL-1β expression, and astrocyte activation, demonstrating that nanoparticle-mediated delivery can enhance the efficacy of anti-inflammatory and neuroprotective interventions within the injured CNS (137). Similarly, ceria-based nanoparticles possess intrinsic reactive oxygen species (ROS)-scavenging activity through reversible redox cycling between Ce^3+^ and Ce^4+^ states. In experimental ischemic stroke models, systemically administered ceria nanoparticles reduced oxidative injury and exerted neuroprotective effects, highlighting the potential value of nanoparticle platforms capable of simultaneously targeting oxidative stress and inflammation (138).

Beyond individual nanoparticle formulations, hybrid nanosystems integrating targeted delivery strategies with controlled-release kinetics are under active development to prolong therapeutic exposure within the CNS microenvironment (139). Such platforms may be particularly valuable for cytokine-directed therapies that require sustained modulation of inflammatory signaling. Although nanoparticle-based delivery has not yet been specifically applied to IL-17A-targeted therapy in TBI, continued advances in nanotechnology may facilitate the translation of cytokine-targeting strategies for CNS inflammatory disorders.

In addition to target selection, the feasibility of Th17/IL-17A-directed interventions in TBI is closely linked to delivery constraints and the broader systemic immune context. Nanoparticle-based delivery systems may help overcome pharmacokinetic barriers and improve CNS exposure of immunomodulatory agents, thereby expanding the therapeutic potential of IL-17A-targeted therapies following brain injury.

### 5.4 Restoring immune homeostasis for IL-17A-targeted therapies

Beyond directly neutralizing IL-17A or inhibiting Th17 differentiation, broader strategies aimed at restoring immune homeostasis have emerged as a complementary framework for modulating post-traumatic neuroinflammation (124). Regulatory T cells (Tregs) are key suppressors of excessive immune activation and can counterbalance Th17-associated inflammatory responses. Experimental stroke studies demonstrated that depletion of Tregs increased infarct volume, worsened neurological outcomes, and enhanced inflammatory-cell activation, whereas Treg-derived IL-10 signaling exerted potent cerebroprotective effects (140). More broadly, Treg and Th17 differentiation are reciprocally regulated, with the cytokine milieu favoring either Foxp3+ regulatory T-cell or IL-17-producing Th17-cell programs (141). These findings support the concept that enhancing regulatory immune mechanisms may help limit pathological inflammation following CNS injury.

Restoration of immune homeostasis may also involve modulation of the gut-immune axis. As discussed above, TBI is associated with persistent alterations in gut microbial composition and intestinal homeostasis (77, 110). Given the established role of the gut microbiota in regulating Th17-cell differentiation and immune balance, microbiome-directed interventions may represent an indirect strategy for modulating IL-17A-associated immune responses after brain injury. Experimental studies have demonstrated that specific commensal bacteria can profoundly influence intestinal Th17-cell development, highlighting the close relationship between microbial composition and type 17 immunity.

In addition, CNS injury induces systemic immune alterations through neurogenic and sympathetic signaling pathways, resulting in widespread changes in peripheral immune function (142). These observations underscore the systemic nature of post-traumatic immune dysregulation and suggest that therapeutic approaches aimed at restoring immune balance may complement direct cytokine-targeting strategies.

Collectively, enhancement of regulatory immune pathways, restoration of gut microbial homeostasis, and normalization of systemic immune responses may provide complementary approaches for modulating IL-17A-associated inflammation after TBI. Given the complexity of post-traumatic immune dysregulation, restoration of immune homeostasis may ultimately prove more effective than targeting individual inflammatory mediators in isolation.

## 6 Emerging directions and context-dependent roles of IL-17A in TBI

Recent advances in neuroimmunology have revealed that IL-17A-associated immune responses involve complex interactions among adaptive immune cells, innate immune populations, glial cells, and peripheral immune organs. These findings suggest that the role of IL-17A in TBI extends beyond a single pro-inflammatory mediator and may influence multiple aspects of post-traumatic neuroimmune regulation (7).

Emerging evidence further suggests that the functional consequences of IL-17A signaling may vary across different stages of injury. During the acute phase, IL-17A may contribute to BBB disruption, inflammatory-cell recruitment, and amplification of local neuroinflammatory responses. In later phases, persistent IL-17A-associated signaling may participate in chronic neuroinflammation, impaired tissue repair, and long-term alterations in neuronal and network function. However, the temporal and cell-specific mechanisms underlying these effects remain incompletely understood.

Importantly, IL-17A is unlikely to function exclusively as a detrimental mediator. As with many inflammatory pathways, its biological effects are likely to be context-dependent and influenced by factors such as injury severity, timing, cellular source, and the surrounding immune microenvironment. Whether Th17/IL-17A responses differ across mild, moderate, and severe TBI remains unclear and warrants further investigation. A more comprehensive understanding of these context-dependent effects will be essential for the rational development of IL-17A-targeted therapeutic strategies.

Taken together, current evidence supports a role for IL-17A as a potentially important regulator of post-traumatic neuroimmune responses. Future studies integrating cellular, molecular, and systems-level approaches will be required to clarify its functions across the acute, subacute, and chronic phases of TBI and to determine whether modulation of the Th17/IL-17A axis can be translated into effective therapeutic interventions.

### 6.1 Longitudinal dynamics of Th17 cells and IL-17A across the TBI spectrum

Although available studies are consistent with increased IL-17A-related signaling during the early inflammatory response after TBI, the long-term temporal dynamics of IL-17A remain poorly understood (7). Most preclinical studies have focused on acute post-injury time points, whereas clinical evidence suggests that alterations in systemic inflammatory profiles may persist for months after injury, with several cytokines, including IL-6 and IL-10, remaining dysregulated across extended follow-up periods (143, 144). Whether IL-17A itself exhibits similar long-term dynamics and contributes to persistent neurological dysfunction remains unclear.

Determining whether sustained IL-17A-associated signaling contributes to chronic neuroinflammation, persistent glial activation, and progressive neurodegenerative processes represents an important research priority (9). Integrating longitudinal cytokine profiling with advanced neuroimaging and cerebrospinal fluid biomarker analyses may help clarify the relationship between immune activity and long-term structural and functional outcomes following TBI (119).

### 6.2 Neuroimmune interactions revealed by multi-omics approaches

Single-cell and spatial transcriptomic studies have provided high-resolution maps of cell-type- and region-specific inflammatory responses after TBI; however, whether IL-17A-associated signaling signatures can be reliably identified within these datasets remains to be systematically investigated (145). These approaches reveal distinct microglial and astrocytic transcriptional states after injury, although direct attribution of specific glial states to preferential IL-17A responsiveness remains limited (146).

Integrating spatial transcriptomic, metabolomic, and other multi-omics datasets with computational analyses may help define relationships between regional inflammatory activity and metabolic heterogeneity after TBI; however, the specific contribution of IL-17A to these processes remains unclear (147). Such datasets can identify cell-type-specific candidate targets, including metabolic enzymes and membrane proteins, that may represent future therapeutic opportunities (148). Whether any of these candidate pathways are directly regulated by IL-17A signaling in TBI remains unknown and requires targeted mechanistic investigation.

### 6.3 Systems-level understanding of peripheral-central immune coupling

TBI triggers systemic immune alterations beyond the CNS that can influence clinical trajectories and recovery (23). IL-17A-related T-helper cell responses have been proposed as one component of the peripheral immune alterations that may influence central inflammatory processes after TBI (23). TBI is associated with disturbances of the gut-brain axis, and gut microbiome composition is known to influence Th17-associated immunity; however, direct evidence that post-TBI dysbiosis drives sustained systemic IL-17A elevation and contributes to ongoing neuroinflammatory processes remains limited (97, 149). TBI can activate sympathetic signaling that modulates bone marrow hematopoiesis and alters myeloid output; whether these hematopoietic changes specifically enrich Th17-associated signatures in circulating immune pools requires further validation (110).

Together, these observations are consistent with a growing recognition that TBI should be viewed as a multi-organ disorder rather than an isolated CNS injury (150). Systems immunology approaches may help identify regulatory hubs linking peripheral immune alterations to central neuroinflammation, with IL-17A-related pathways representing one candidate axis for such integrative analyses (151).

### 6.4 Reconciling the dual roles of IL-17A in injury and repair

IL-17A is widely recognized as a pro-inflammatory cytokine capable of amplifying inflammatory signaling across multiple tissues, with growing evidence indicating comparable pro-inflammatory effects within the CNS (53). However, injury responses after TBI are increasingly conceptualized as evolving biological processes rather than static events, with immune mechanisms changing across different phases of injury (124). Within the dynamic, stage-dependent framework of TBI (124), it remains unclear whether IL-17A signaling exerts divergent effects across cellular contexts and time windows. Given the diversity of IL-17-producing immune populations described across inflammatory settings (53), dissecting their relative contributions may help clarify context-dependent immune effects after CNS injury. Such considerations are consistent with the broader view that TBI represents a dynamic, stage-dependent disease process (124), suggesting that cautious, stage-aware immunomodulatory strategies may be preferable to indiscriminate cytokine suppression.

### 6.5 Translational and clinical roadmap for IL-17A-targeted therapies

The availability of clinically approved IL-17A inhibitors for autoimmune diseases underscores the clinical feasibility of targeting this pathway and may lower the translational barrier for evaluating related strategies in TBI (152). However, based on prior experience from TBI clinical trials, successful clinical translation is likely to depend on careful optimization of treatment windows, dosing strategies, and patient stratification (153).

Building on prior studies of fluid and molecular biomarkers in TBI (154, 155), biomarker-based approaches, including circulating cytokine profiles and other indicators of inflammatory pathway activation, may help improve patient stratification and therapeutic timing in future targeted immunomodulatory studies. Moreover, combining IL-17A pathway modulation with complementary immunomodulatory or delivery-based approaches may represent a strategy for more broadly influencing neuroimmune interactions after TBI.

As the field advances, integrating mechanistic insights from preclinical studies with biomarker-guided clinical investigation will be important for determining whether targeted interventions, including those directed at the IL-17A pathway, can influence long-term outcomes after TBI (124).

## 7 Conclusion

Post-traumatic neuroinflammation involves complex immune responses, and Th17/IL-17A-related mechanisms have been discussed within this broader immunological context in TBI. However, their specific contribution to linking early immune activation with longer-term neurological outcomes remains incompletely defined (7, 20, 100, 156). Experimental studies indicate that IL-17A has been implicated in several components of secondary injury cascades, including BBB dysfunction, glial activation, and neuronal signaling, supporting its consideration as a potential therapeutic target (51, 100, 101, 113).

Viewing TBI as a dynamic and evolving disease process (124), broader immunomodulatory strategies have been proposed as a conceptual therapeutic framework (7, 125). Within this context, cytokine-targeted approaches, including those involving IL-17A pathways, have emerged as candidates of interest, although their effective clinical translation and integration with advanced delivery platforms remain important areas for future investigation (20, 100). Consistent with prior experience in TBI research, integrating mechanistic insights with rigorous clinical trial design will remain essential for evaluating the translational potential of emerging therapeutic strategies (153, 157).

Taken together, current evidence supports a conceptual framework in which Th17/IL-17A signaling may contribute to the interplay between early neuroimmune activation, neurovascular dysfunction, and longer-term neuropathological sequelae following TBI. Future studies should continue to address several unresolved questions regarding Th17/IL-17A biology in TBI. In particular, it remains unclear whether Th17/IL-17A responses differ across mild, moderate, and severe TBI, and clarifying this issue may provide additional insights into disease progression and the development of precision immunotherapeutic strategies. Ultimately, continued investigation into the diverse roles of Th17/IL-17A signaling in TBI will further advance our understanding of post-traumatic neuroimmune regulation and support the development of more precise and effective immunotherapeutic strategies.
